# Associations Between Ancillary Body Movements and Acoustic Parameters of Pitch, Dynamics and Timbre in Clarinet Playing

**DOI:** 10.3389/fpsyg.2022.885970

**Published:** 2022-07-13

**Authors:** Manfred Nusseck, Isabella Czedik-Eysenberg, Claudia Spahn, Christoph Reuter

**Affiliations:** ^1^Freiburg Center for Research and Teaching in Music, Freiburg Institute for Musicians’ Medicine, University of Music Freiburg, Medical Faculty of the Albert-Ludwigs-University Freiburg, Freiburg, Germany; ^2^Musicological Department, University of Vienna, Vienna, Austria; ^3^Music Technology and Digital Musicology Lab, Institute for Musicology and Music Pedagogy, Osnabrück University, Osnabrück, Germany

**Keywords:** body movements, motion capture, signal analysis, music information retrieval, expression

## Abstract

When playing an instrument, there are two main categories of body movements: instrumental movements, which are necessary for the sound production, and ancillary movements, which are associated with individual musical intentions and expressions. In this study, the particular purpose of ancillary movements of clarinet player was investigated especially in respect to how these movements were related to the musical structure of the piece and to specific audio parameters. 3D motion capture data of 19 clarinet players performing the same piece were analyzed regarding common motion patterns during the performance and in accordance with acoustic features related to pitch, dynamics (RMS energy) and timbre (spectral centroid and flux). A focus of the body movements was on the arms and the knees. The results showed that there were certain motion patterns performed by the players depending on specific musical structures. When playing a melodic part, the players often did so by bending their knees. At musical transitions, however, the knees were mainly stretched. Similarly, arm movements were more pronounced during playing melodious parts. At transitions, the arms were put closer to the torso. Considering the connection with the acoustics, a larger range of knee motions was correlated with a larger variation of the timbre. Moreover, at specific moments during the performance, when some players strongly bent their knees or lifted the arms, the RMS energy of the signal was significantly higher. The correlations of the body movements and the acoustic features showed that some players synchronized their movements with particular audio parameters more than others did. In summary, the ancillary movements of the clarinetists pursued both musical expressive intentions and physiologically necessary movements and tended to be performed with individual differences in terms of visual and auditory expression.

## Introduction

Body movements are essential for playing an instrument. Investigating body movements in musicians became a growing research topic in the last few decades (Godøy and Leman, 2010; Gritten and King, 2016; Wöllner, 2017; Jensenius, 2018). Playing a traditional instrument requires physical activities that are inseparable from the sound production (Wanderley and Battier, 2000). However, there are also movements that are not directly involved in the sound production. Hence, the functional aspects of musician’s motions can generally be classified into two groups: instrumental movements, which are necessary to produce the sound on the instrument, and body movements, which do not directly generate sound, the so-called ancillary movements (Cadoz and Wanderley, 2000; Jensenius et al., 2010; Nusseck et al., 2018). Where the sound-producing movements are strictly bound to the instrument, ancillary movements are more open and convey individual intentions and expressions as well as personnel characteristics of the musician (Godøy and Leman, 2010). In examining performers’ expressive movements, viewers were able to identify which emotions musicians intended to express while playing and the extent of their expressivity by looking at video recordings or point-light displays of musicians (Dahl and Friberg, 2007; Nusseck and Wanderley, 2009; Davidson and Broughton, 2016). Main body areas of ancillary motions are head movements, facial expressions, and side-to-side swaying (Demos et al., 2018). Furthermore, ancillary movements can additionally provide communicative aspects such as giving a cue to other musicians and making eye-contact between musicians or to the audience (Jensenius et al., 2010; Coorevits et al., 2020). In a music ensemble, anterior–posterior body sway has been found to reflect joint emotional expressions (Chang et al., 2019).

The different kinds of body movements usually occur simultaneously during musical performances (Dahl et al., 2010). They serve multiple purposes and form a complex system that changes with respect to the musical performance. For example, the execution of ancillary movements differs when playing with different emotional states and when playing solo compared to playing in an ensemble (Davidson, 2011; Glowinski et al., 2013).

When playing the same piece, instrumental movements are habitually very similar, since the notes have to be played on the instrument. Likewise, ancillary movements also sometimes follow similar patterns and are often aligned with rhythmic patterns and occur at musically important points (Wanderley et al., 2005; Davidson, 2007). For instance, all movements usually stop at the end of a phrase and at a fermata to emphasize the melodic paragraph. This indicates that beside the individual use of ancillary body motion to express certain personal musical intentions there are also common movement patterns related to key musical moments. Considering different expressive performances of the same piano piece, increased movements in an exaggerated performance seemed to take place at similar musical moments (Thompson and Luck, 2012). The authors conclude that the performance of expressive movements is influenced by the musical structure.

For trombone players, it was found that body swaying seemed to be related to certain musical structures and was performed rather similarly by the same performers (Demos et al., 2018). However, it appears that ancillary movements are less consistent across pieces and players, indicating that musicians seem to use different movements in different musical contexts, even within the same performance (Davidson, 2012). As music is produced in the moment, performances of the same piece can differ considerably (Juslin and Timmers, 2010).

For clarinet players, the movement of the bell was used as indicator of music-related body motion and showed certain consistencies between performances of the same piece (Wanderley et al., 2005; Vines et al., 2006). The clarinetists were asked to perform with three different degrees of expression, i.e., immobile, standard and exaggerated, and the movements of the bell were rather similar for each player, but varied between the expressivities with larger motion ranges in the exaggerated performances. In a different study, clarinetists’ ancillary movements were quite similar in terms of expressive duration variations for certain melodic phrasings and harmonic transitions (Teixeira et al., 2015). They also showed that recurrent motion patterns of the clarinet bell occurred at specific musical passages, e.g., at the end of the piece. In a solo, however, clarinetists usually stand, which gives them more freedom of movement than just the motion of the bell, i.e., to sway and bend their knees.

**Figure 1:**
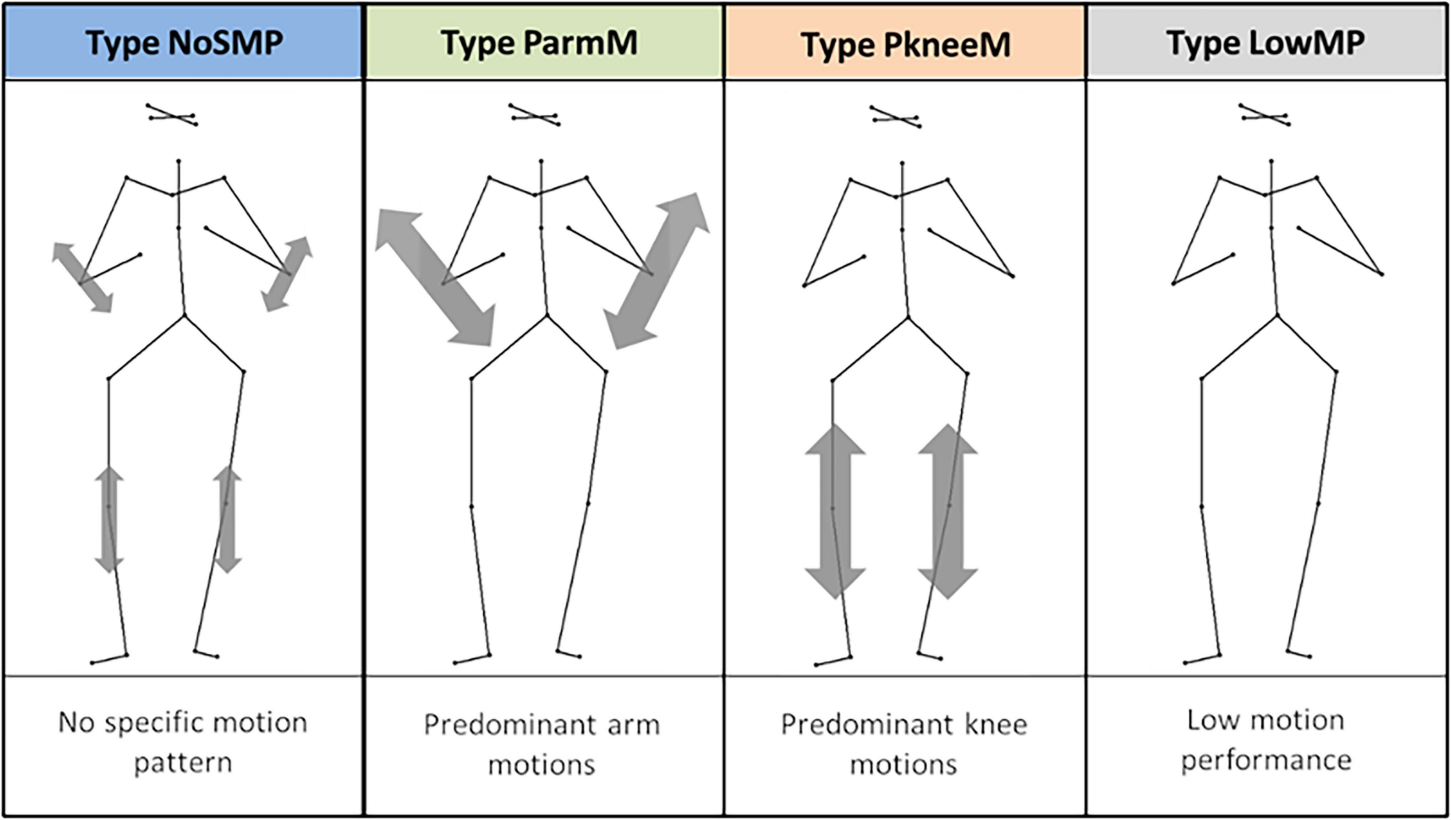
Motion types of clarinetists found in Weiss et al. (2018).

In a previous study, ancillary full body movements of clarinetists were analyzed to identify commonalities in movement patterns by using 3D motion capture (Weiss et al., 2018). Angular movements of several body parts of 22 clarinetists playing five different solo pieces were calculated. A cluster analysis on the variances of these angles revealed four different motion types with specific motion characteristics: (1) with predominant knee motions (PkneeM), where players preferably performed with large knee bending, (2) with predominant arm motions (ParmM), where players particularly moved the arms, (3) with no specific prominent motion pattern (NoSMP), where players used both arms and knees to a lesser extent than in case of the first two motion types, and (4) with an overall low motion performance (LowMP), where players performed with lower motion amplitudes in all body regions. Figure 1 illustrates the different motion types with their main movement areas on a point light display. The motion types distributed across players and performances of 34% the NoSMP, 13% the PkneeM, 14% the ParmM and 39% the LowMP type. In summary, the study identified two main movement areas of clarinetists: the arms in the upper body segment and the knees in the lower body segment. These areas were mainly corresponding to expressive intentions and have also been observed in previous studies (Wanderley et al., 2005; Davidson, 2012).

In multimodal – audio and visual – performances, it has been shown that the observed musician’s movements considerably contribute to the perception of the performance (Vines et al., 2006; Nusseck and Wanderley, 2009; Platz and Kopiez, 2012; Tsay, 2013). Those studies highlight the importance of the visual component in musical performances providing a source of information that can change the judgment of the performance. By using the above mentioned motion types of clarinetists in a perceptual experiment, participants were asked to rate the musical performance and expressivity when seeing the motions of the different types but hearing the same audio track (Weiss et al., 2018). The results showed that the motion types with a predominant movement area, i.e., the knees or the arms, were rated higher in their expressivity than the players with no specific motion pattern. The lowest ratings were given to the players with overall low motion performance. In summary, the findings confirmed the influence of visual movements on the perception of the musical performance especially regarding the communication of expressive elements.

Despite visual expressiveness, an important source of expressive intentions in music is provided by the sound. For that, musicians associate changes in acoustic signals, such as dynamics, timing, tempo, loudness, with certain articulations (Gabrielsson and Juslin, 2003; Juslin and Timmers, 2010). However, since body motions are inseparable from the sound, certain acoustic features might be connected with particular movement characteristics (Godøy and Leman, 2010). Musicians’ movements contain constitutive elements of the performance, which can also contribute on the acoustic level. In a study examining listeners’ responses to static or moving orchestral musicians playing solo, it was found that the moving musicians were easily identifiable (Ackermann et al., 2019).

Ancillary movements of musicians with expressive intentions were also found to be associated with specific acoustic changes. When measuring the acoustic energy (RMS) in piano performances of the same piece played with different levels of expression (immobile, with low expressivity, with normal expressivity, and exaggerated), most dynamic variations were found in the exaggerated condition, while the other conditions remained relatively equal (Thompson and Luck, 2012). By analyzing the movements of the clarinet bell, Teixeira et al. (2018) found similar motion patterns linked with particular acoustic variations in loudness and timbre at specific musical positions such as melodic phrasings and harmonic and dynamic transitions. They concluded that certain musical structures lead to the appearance of specific ancillary movements. Accordingly, those movements seem to be used to support acoustic expressions.

Aside from the clarinet bell, it may be possible that other common motion patterns while playing can be related to the acoustic expression. As a wind instrument, the clarinet is connected to the mouth and limited in its range of motion. Similarly, the hands are fixed to the instrument. Moving the clarinet is therefore dependent on movements in other body areas such as the arms, the torso and more indirectly also the legs. Thus, additional body movements besides the clarinet bell should be focused on in investigations for acoustic correlations. For example, it is possible that the bending of the knees is associated not only with a visual expression, but also with an acoustic change, for instance with an increase in dynamic. Furthermore, it remained unclear how ancillary movements are related with acoustic features in between certain expressive musical positions.

In this study, following the particular motion types of clarinetists described above (Weiss et al., 2018), the use of ancillary movements focusing on the arms and the knees was examined in more detail in terms of how players moved according to the musical structure of the piece and regarding changes in acoustics features. This leads to two major areas of questions:

1. **Visual aspects:** Do players perform similar ancillary movements following the musical phrasing? For this purpose, the arm and knee angular movements were analyzed for the occurrence of particular common motion patterns across players. Those patterns were then related to musical structural elements, such as transitions and melodic sections.
2. **Acoustic aspects:** Are ancillary movements related to certain acoustic features? Here, acoustic changes were analyzed in conjunction with angular movements in the arms and knees. For that, angular peaks in movement were selected and analyzed for differences in the acoustic parameters. In addition, the overall angular movement variability in the arms and the knees was tested for correlations with the variance of the acoustic parameters. It is expected that due to the relation of ancillary movements with expressive intentions, larger ranges of angular movements may be connected with higher acoustic variations.

Using the motion capture data of the clarinetists, ancillary movements of the arms and the knees were analyzed in relation to their appearance during playing concerning the musical structures of a certain piece and in conjunction with particular acoustic parameters. For that, the analyses were performed on the angle values in the arms and the knees first of all players and secondly separated by motion types. Examining the differences between the motion types is of particular interest because there are players with large ranges of angular motions in either arms or knees and players with very little motion at all. Differences can be discussed in accordance to the motion types and therefore to individual styles of clarinet playing.

The analysis was divided into three parts: first, with a focus on visual movements, i.e., body movements in relation to the musical structure; second, with a focus on acoustic parameters such as dynamics and timbre in relation to the musical structure; and third, with a multimodal focus on the relationships between visual movements and acoustic changes.

## Materials and Methods

### Clarinet Players

The recordings of clarinet players reported in Weiss et al. (2018) have been used. Of the original 22 clarinet players, the data set for this study included 19 players who provided complete sets of both motion and audio data. Nine players were female and ten players were male. The mean age was 32.7 years (SD 12.8 years) and the average duration of playing the clarinet was 22.5 years (SD 12.3 years). Regarding the motion types, five players were in the NoSMP type, four players in the ParmM Type, three players in the PkneeM type and seven in the LowMP type.

The players were originally asked to perform five different solo clarinet pieces lasting about 20 to 60 s. The clarinetists played in standing position and were not allowed to walk around or to take steps. They played each piece once. In case of misplays, the recording was repeated. The sheet music was provided on a music stand about 60 cm in front of them. All players confirmed their written consent to participate in the study.

### Recording Setup

An optical 3D motion capture system with four calibrated digital video cameras was used to record the visual movements. The sampling rate was 50 frames per second. Twenty-two reflective markers were placed at central joints and body positions of the players to obtain a whole-body representation (Figure 1). There were four markers on the head, two on the shoulders, two on each arm, three on the torso, three on the hip, and three on each leg. The videos were processed with the Templo Software (Contemplas) and the 3D marker positions were digitized by using Peak Motus 10 (Vicon).

Specific body angles were calculated by using particular marker coordinates. The values of the range of angular motions were analyzed to classify the different motion types (see above, Figure 1). The method used to characterize the types of motion is presented in more detail in Weiss et al. (2018).

The sound was recorded with a digital audio recorder (Zoom H4N). It was placed on a stand at 1 m height and approximately 2 m in front of the players. A stereo WAV-File with 44.1 kHz sample rate was used. Synchronization between the motion capture and the audio recordings was established by a simultaneous visual and acoustic gesture of the players immediately before each start of the performance. In post-processing, the video and the sound were merged based on this signal.

### The Music Piece

In this study, the piece in which the different motion types of clarinettists were most clearly distinguishable was chosen from the recorded pieces. The piece was Mendelssohn’s 3^rd^ Symphony (“Scottish Symphony,” Op. 56, the first 24 bars of the 2nd movement “Vivace” in A minor). The score of this piece can be found in the Supplementary Material. The tempo was given to the players by a metronome (126 bpm) directly before the performance but they did not perform with the metronome. The mean duration of the piece was 24 s.

The melodic structure of the piece consists of six phrases. The first phrase frames the first eight measures (with upbeat) and presents the main melody. The next eight measures repeat the complete previous melody. Unlike the more melodic preceding parts, which contain several sixteenth notes, the next four measures contain a sustained note. The final phrase repeats the previous four measures with a slightly altered harmonic motion.

**Figure 2:**
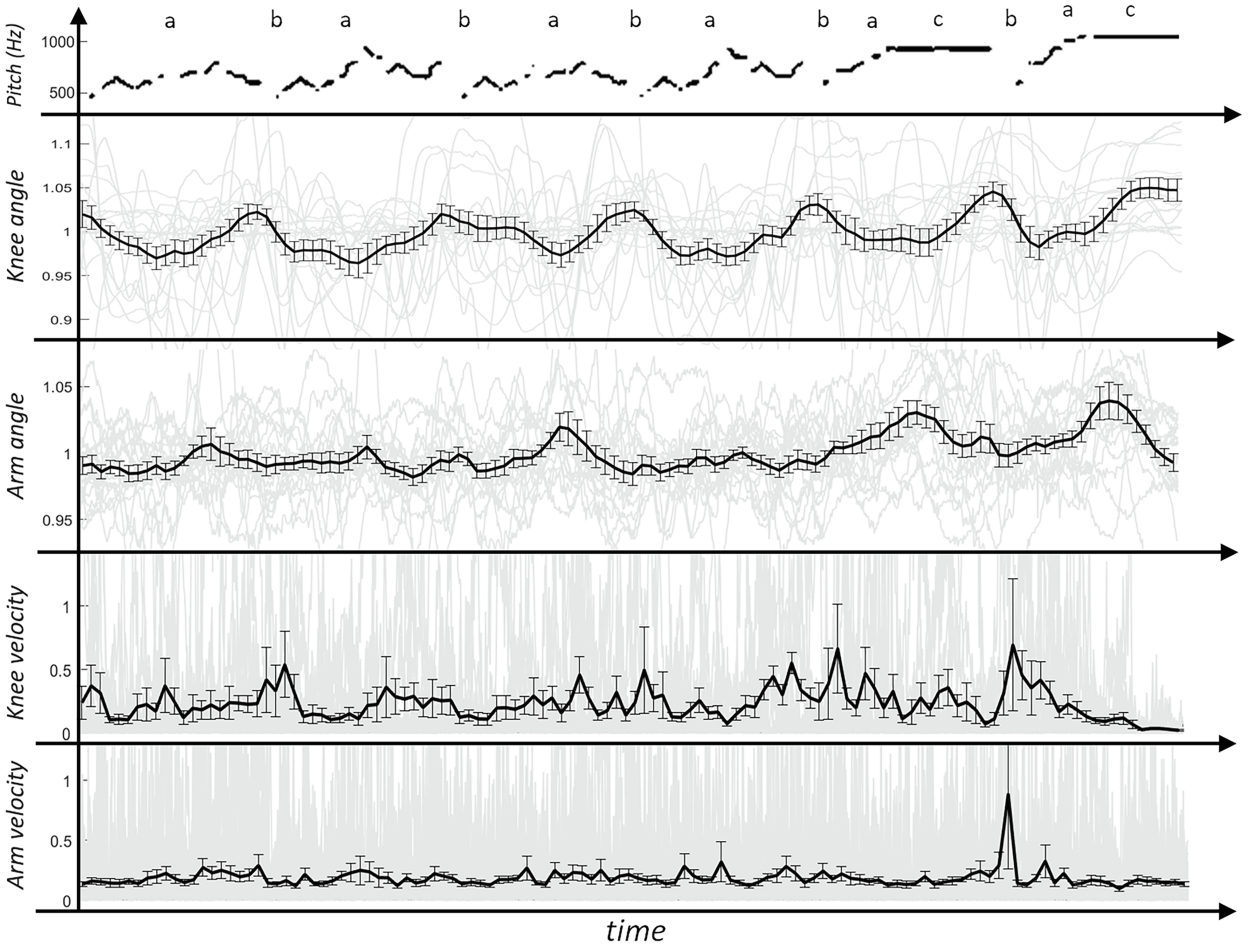
Graphs of the visual movements of the knees and the arms (top row: schematic of the pitch; second and third row: normalized motion of the angle; fourth and fifth row: velocities in 1/50s; gray lines: individual values of the players; thick black line: mean value across all players; error bar: standard error of the mean).

**Figure 3:**
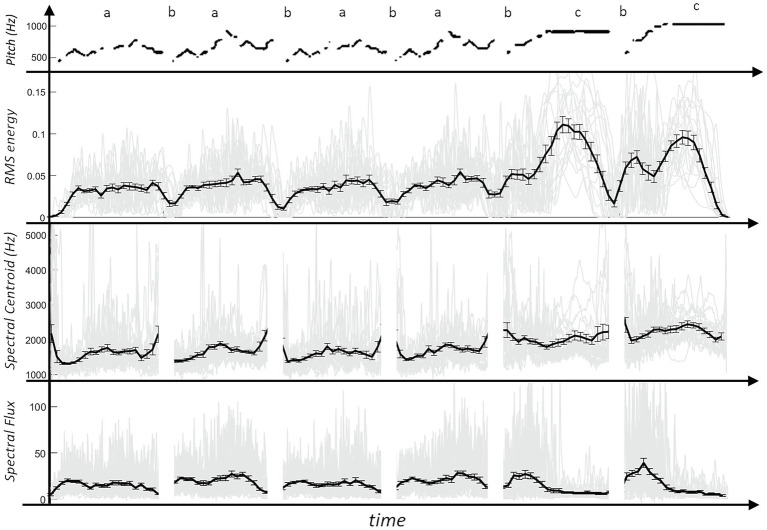
Graphs of the four acoustic parameters (top row: schematic of the pitch; gray lines: individual values of the players; thick black line: mean value across all players; error bar: standard error of the mean).

It is possible to inhale at the transitions of each phrase, i.e., between the main melody parts, before the two final phrases and between the two final phrases, what most of the players did. A schematic representation of the melodic pitch can be seen in the top line of Figures 2, 3.

### Data Analysis

#### Visual Movements

The movement analyses included a focus on specific areas of interest for clarinet playing. Weiss et al. (2018) found that the main movements were in the arms and legs. Shoulders, the instrument or side-to-side motions did not show significant common patterns between players. Therefore, the main analyses of the visual movements in this study focused on arm and knee movements, especially angular movements.

The knee angle was calculated from the combination of the foot, the knee and the hip marker. A high value means an extended leg and the lower the angle, the more the knees are bent. Limited by anatomy, the value cannot be higher than 180 degrees and no angle was found below 100 degrees. Both knee angles were highly correlated (*r* > 0.9). Thus, only the right knee angle was used for the analyses.

Since the arm angle was formed from the markers of the elbow, shoulder and neck, a higher value represents a more raised arm with an opening in the armpit. Anatomically, the value ranged from 90 to 180 degrees, with an arm very close to the trunk being about 90 degrees and an arm raised almost horizontally being about 180 degrees. Both arm angles correlated highly with each other (*r* > 0.8). Since the right thumb has a holding function of the instrument, this resulted in a slightly reduced range of motion of the right arm (Weiss et al., 2018). Therefore, only the left arm angle was used for the analyses.

Due to the anatomical differences between the players, the raw data of each player were normalized by dividing the angular values by the mean value of the angle. This procedure yields values around one. Larger angles are above one and smaller angles are below one. The resulting value has no unit.

In addition, the velocity of the angles was calculated for both the arm and knee angles. For that, the difference of two successive angle values was calculated. This therefore corresponds to the angular change over time, i.e., a fiftieth part of a second. The velocities were calculated as absolute values to obtain distinct peak velocities. The value is zero if the movement had stopped at reversal points.

#### Acoustic Parameters

Following Teixeira et al. (2018), the sound analyses of the clarinet recordings focused on the musical parameters of dynamics, timbre and additionally the pitch trajectory. Specifically, the pitch represents the acoustic realization of the notes seen in the score (Supplemental Material), i.e., the higher the note played, the higher the detected fundamental frequency at a specific time frame. For capturing dynamic changes within the performance, the RMS energy of the audio signal was analyzed over time, which was calculated as the root average of the squared amplitude of the signal. As a descriptor for the timbral brightness, the spectral centroid (i.e., the mean of the spectral distribution in Hz) was included. Additionally, the spectral flux was included in order to quantify timbral change and fluctuations. Similar to the calculation of the angular movements’ velocity, the spectral flux determines the distance of the power spectrum between two successive time frames. Its value increases with the amount of spectral fluctuations present.

To extract those audio features, the MIRtoolbox 1.6.1. (Lartillot et al., 2008) for MATLAB (R2020a) was used. Acoustic features were extracted using half-overlapping time frames with a length of 0.05 s. In order to obtain a data set that can be correlated with the visual motion data set, the acoustic data were sampled to match the resolution of the motion capturing data. Both visual and acoustic data were equally reduced to 120 data points for each recording.

Since the spectral parameters were also collected at moments when the clarinetists did not play, i.e., during breathing, the values of these features would measure the background noise causing value artifacts. Therefore, a kind of “noise-gate” was used to exclude those pauses from the analysis. For that, when the RMS energy value was less than 0.02, the values of the collected timbre features at this frame were excluded from the analysis. This setting was also applied when determining the mean values of the timbre features.

#### Combined Visual and Acoustic Analysis

For the combined analysis of both visual and audio, three different approaches were taken. First, the mean values and variances of the acoustic parameters were compared between the different motion types.

**Figure 4:**
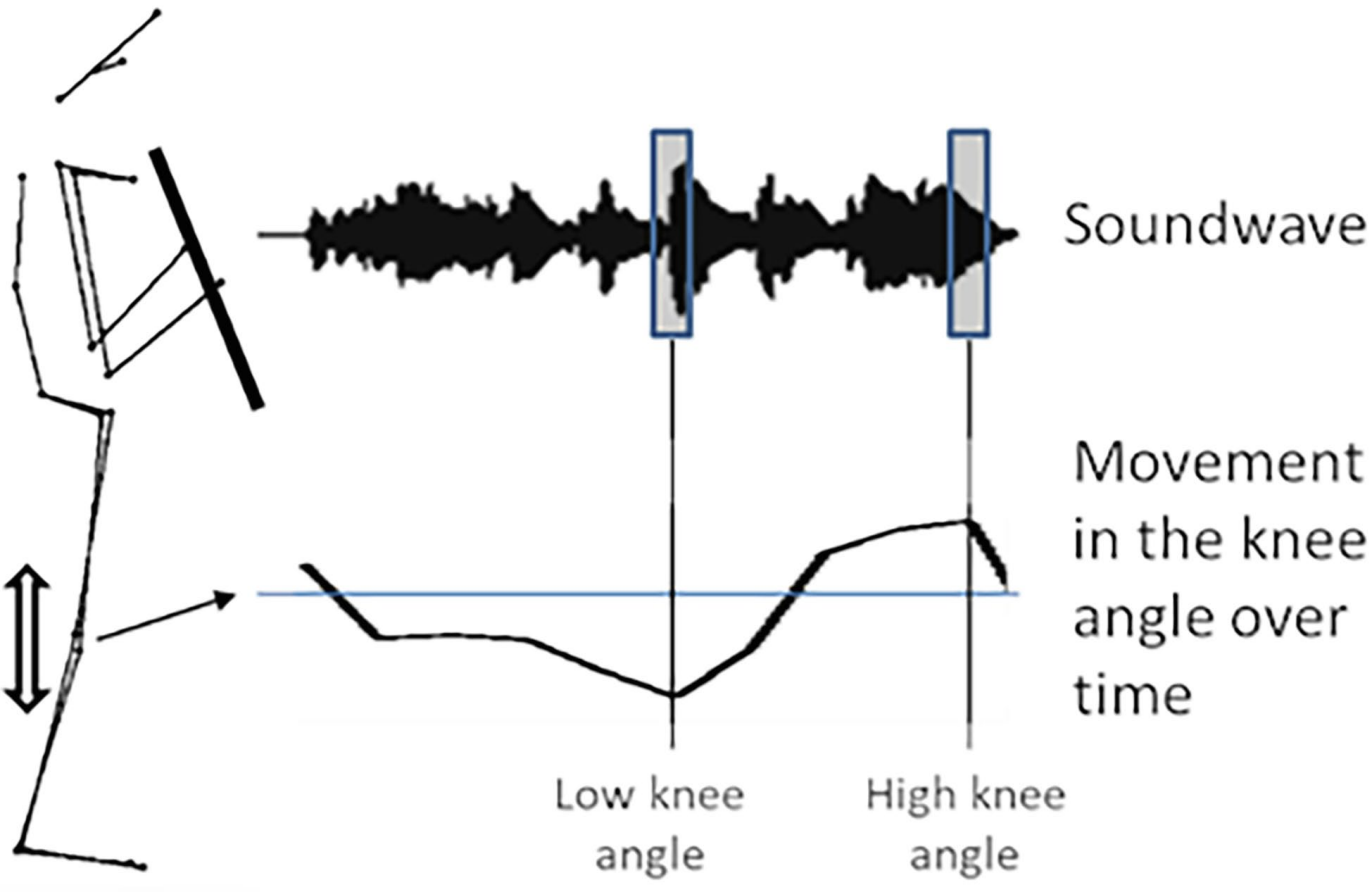
Schematic of the multimodal analysis of motion peaks with sound parameters by the example of the knee movements.

In a second approach of the analysis, the sound parameters were calculated at specific points of interest in the motion of each player. For that, the motion curves of the angles in the knees and arms were examined for local maxima and minima, i.e., reversal points. A schematic figure of this analysis approach is provided in Figure 4. To this end, automatic peak detection was performed in MATLAB (R2020a) using the function “findpeaks” to identify the turning points in the angular movement trajectories of the arms and knees. At those peak points (including the 10 surrounding time frames), the mean values of the acoustic parameters were measured. During the piece, these peaks occurred frequently, but differed in their total number between the players. Since the movements of the angles are normalized, the mean acoustic values at the two extreme points (lowest angle and highest angle) were compared with each other across all players.

In the third approach, for an even more comprehensive player-specific analysis, correlations were calculated between the 3D motion capture data and the synchronized audio features for each player (P1-P19). Thus, certain relations between body movements and audio features could be evaluated in detail for each individual player categorized by the different motion types.

### Statistics

For the statistical analyses SPSS (Version 28, Armonk, NY: IBM Corp.) was used. Descriptive statistics were calculated for parametric variables including mean values and standard deviations (SD). Chi-square (*χ*^2^) tests were performed for comparisons of non-parametric variables. Parametric comparisons have been calculated with analysis of variances (ANOVA). When the ANOVA was significant with more than two variables, *post hoc* analyses with Tukey HSD (honestly significant difference) correction were performed. Comparisons between the marker positions within the piece has been performed with a paired sample t-test. Relationships between motion angles and audio features were further investigated for each player individually *via* a correlational analysis. Significant correlations above Pearson’s *r* = 0.25 were considered medium, above *r* = 0.5 high correlations (Cohen, 1988). The level of statistical significance was set at *p* = 0.05. However, considering possible multi-testing problems due to the high number of individual combinations, only medium correlations and above (>0.25) were considered for interpretation.

## Results

### Movement Analysis

Figure 2 shows the mean angular movement values of the knees and arms. For both the movement values and the velocities, the individual values of the players (gray) and the mean value of all players (black) are plotted. To perform a comparative analysis, certain positions were selected and marked. These were the positions during the melody (marked with an “a”) and when breathing in (“b”). A third position was chosen during the playing of the prolonged notes in the last two phrases (“c”). For the analysis, the mean values at each marked positions were calculated across players and across the different places in the piece.

In Figure 2, it can be seen that the knees were flexed while playing the melody (“a”) and at the transitions between the phrases (“b”) the legs were stretched to provide a more upright body posture. The comparison between the mean knee angle values at both positions (“a” and “b”) showed a significant difference [*t*(18) = 3.14, *p* = 0.003] with lower values at “a.”

Similarly, the arms were in a rather neutral and aligned position between the phrases (“b”) and during the melody (slightly after each “a”), the arms moved up. The comparison analysis between the mean values at these two positions showed a significant difference [*t*(18) = 1.78, *p* = 0.045] with lower values at “b.” In the last two phrases, when playing the prolonged notes (“c”), the arms were lifted to the strongest degree. The analyses between the position “c” with “a” and “b” yielded a significant effect with significantly higher values at “c” [c-a: *t*(18) = 3.17, *p* = 0.003; c-b: *t*(18) = 3.39, *p* = 0.002].

The analysis of variance performed on the mean values of the velocities in the arms and in the knees between the marked positions yielded no significant effects [*t*(18) < 1.0]. This indicates that no systematic pattern was found for the velocities during the performance. The angular movements of the arms and the knees were rather unvarying and showed only some individual peaks.

### Acoustic Analysis

For the acoustic analysis, three audio parameters had been selected. The RMS energy was chosen to capture dynamic changes. The spectral centroid as well as the spectral flux were chosen to represent timbral aspects. Similar to the visual movements, comparisons of the mean audio feature values between the marked positions were performed. Figure 3 shows the acoustic parameters over the course of the performance. The graphs illustrate when the players inhaled (marked with a “b”) with low RMS energy at these moments. At the same time, the spectral features would change drastically. Therefore, their values were removed during those positions (“b”). The analysis of the mean RMS energy values between the marked positions showed a significant effect with the lowest values at “b” [a-b: *t*(18) = 3.97, *p* < 0.001, b-c: *t*(18) = 11.64, *p* < 0.001]. While the first four phrases show a very similar pattern concerning the signal’s RMS energy, in the last two phrases, the prolonged notes (“c”) were played with significantly higher dynamics than in the previous phrases at “a” [*t*(18) = 8.16, *p* < 0.001].

The spectral centroid and the spectral flux were relatively stable over the piece. However, in the last two phrases (“c”), the mean spectral centroid was significantly higher [*t*(18) = 4.42, *p* < 0.001] than in the previous phrases (“a”). The spectral flux showed two peaks at the beginning of the two last phrases (between “b” and “c”) when there were dense note onsets with a significant difference to the previous phrases [*t*(18) = 3.99, *p* < 0.001]. The spectral flux was also significantly lower during the sustained notes in the last two phrases (“c”) compared to the previous phrases [*t*(18) = 3.87, *p* < 0.001].

### Visual and Acoustic Analysis

#### Comparisons Between the Motion Types

**Figure 5:**
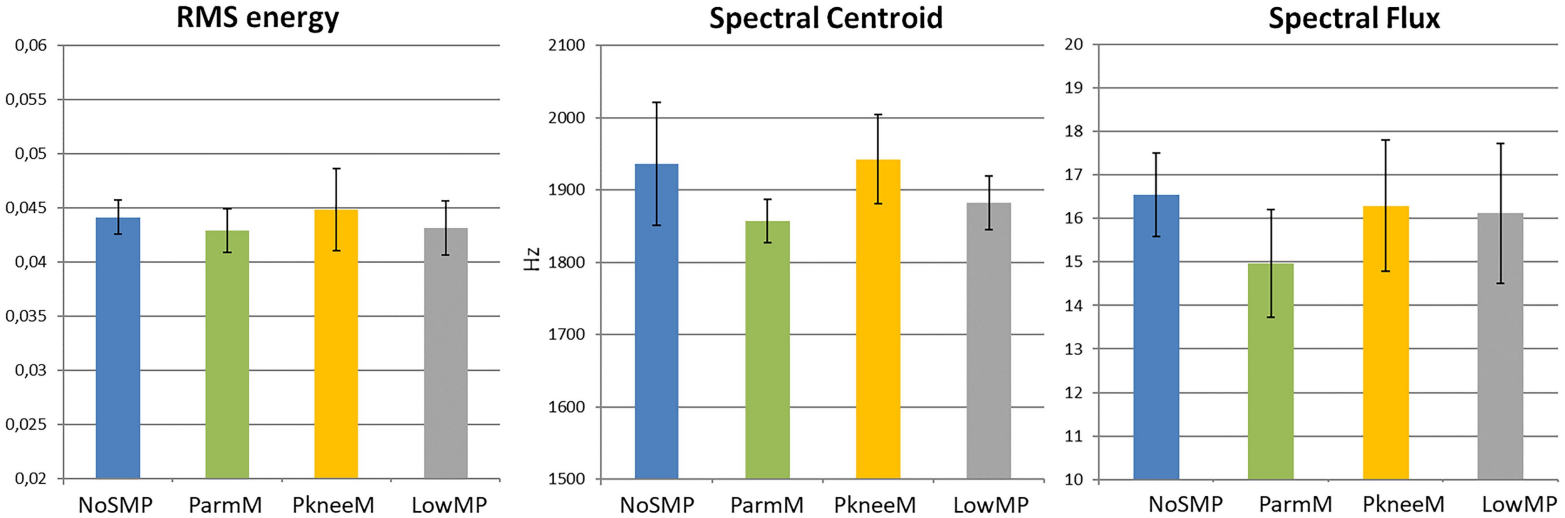
Mean values of the acoustic parameters by motion type (Abbreviations of the motion types see Figure 1; error bar: standard error of the mean).

For analyzing the relations between visual movements and acoustic parameters, statistical comparisons of the acoustic parameters between the motion types were performed. Overall, the analyses of variance showed that there were no significant differences in any of the acoustic parameters [*F*(3,15) < 1.0]. In Figure 5, the mean values of the three acoustic parameters, i.e., RMS energy, spectral centroid and spectral flux are shown by motion type.

#### Movement Peak Analysis

**Figure 6:**
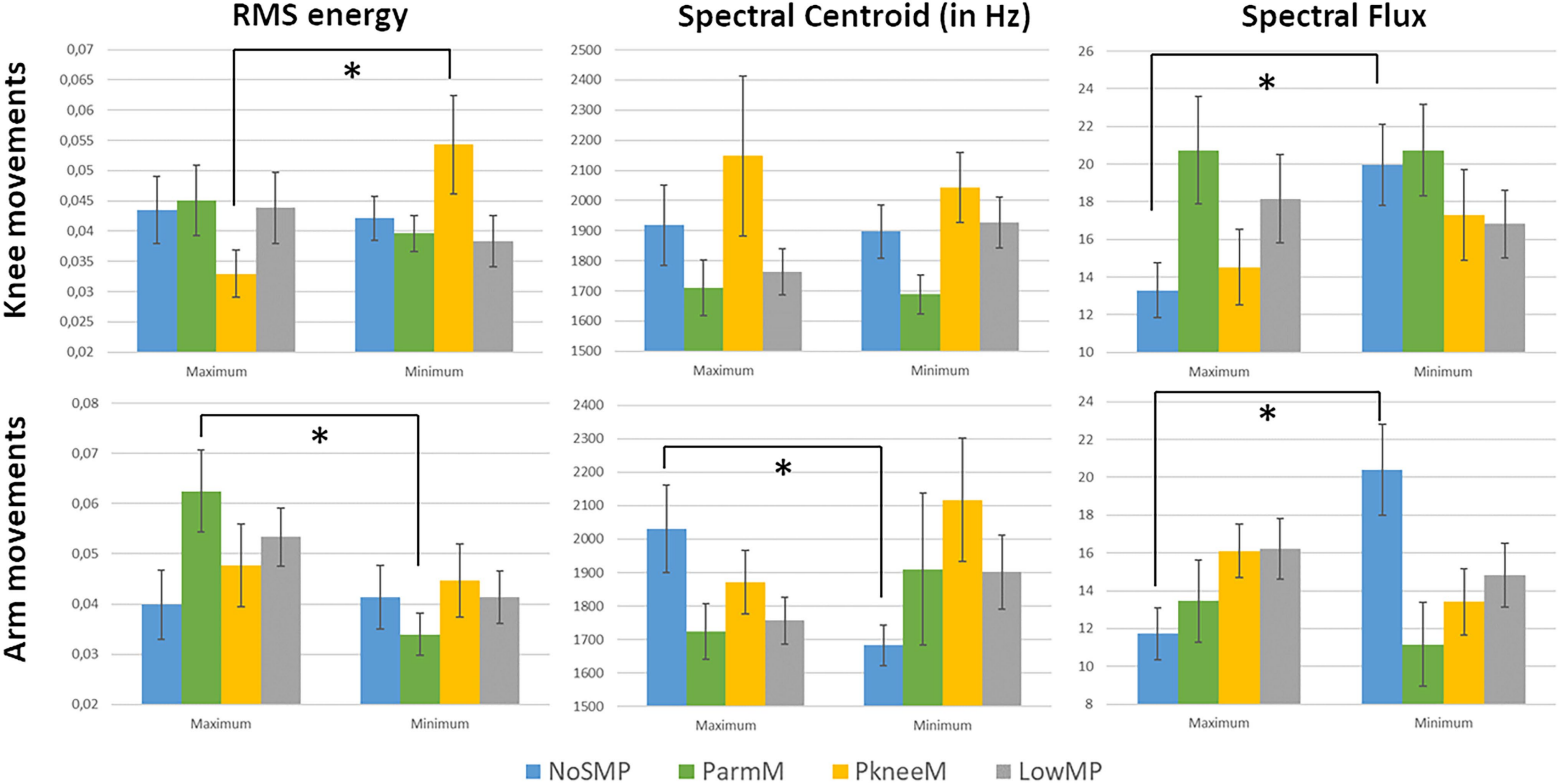
Mean values of the RMS energy, the spectral centroid and the spectral flux by motion type (Abbreviations of the motion types see Figure 1) and movement peaks (maximum: high angular peaks; minimum: low angular peaks; error bar: standard error of the mean; ^*^p < 0.05).

For the analysis of the acoustic parameters at the minima and maxima of the angle curves, the three acoustic parameters of the RMS energy, the spectral centroid and the spectral flux were considered. Figure 6 shows the mean values of each acoustic parameter for high angular points (maximum) and low angular points (minimum). The multivariate analysis of the three acoustic parameters with types and peaks yielded no significant main effects for the knee movements. However, going into more detail, there was a significant difference in the mean RMS energy value of the PkneeM players between maxima and minima of the knee movements [*F*(1,35) = 5.1, *p* = 0.032] with higher values at points of low knee angles, i.e., when the knees were flexed. For the mean values of the spectral flux, the NoSMP Players performed with significantly higher values when they bent their knees [*F*(1,69) = 6.1, *p* = 0.016].

In case of the arm movements, the multivariate analysis showed a significant main effect of peaks in the RMS energy [*F*(3,202) = 4.4, *p* = 0.039] with higher mean values across all motion types at points of high arm angles (maximum), i.e., when the elbows were lifted. The ParmM players performed with different dynamic intensity depending on the arm position. The RMS energy values were significantly different between the maximum and minimum [*F*(1,29) = 9.2, *p* = 0.005] with higher values at points of higher arms angles (maximum), i.e., with spread arms.

There were significant interaction effects of type and peaks for the spectral centroid [*F*(3,202) = 2.8, *p* = 0.038] and for the spectral flux [*F*(3,202) = 4.3, *p* = 0.006] in the arm movements. Especially the players of the NoSMP type showed significant differences in the spectral centroid [*F*(1,58) = 5.6, *p* = 0.021], with higher values when the arms were lifted, and respectively, in the spectral flux [*F*(1,58) = 10.1, *p* = 0.002], with higher values with arms close to the torso.

#### Correlations

It was assumed that a greater range of motion might also be accompanied by greater variability in dynamics or timbre. For that, the correlations between the variances of the motions in the arms and knees with the variances in the acoustic parameters were calculated across all players. In this analysis, the pitch was also included. The correlation coefficients are shown in Table 1. Only the correlation of the variances in the knee motions and variances in the spectral centroid was found significant (*r* = 0.49, *p* < 0.01), indicating that a larger range of motion in the knees was associated with higher variations in the timbre.

**Table 1:** Correlation coefficients with significances between the variances in the motion areas and the acoustic parameters across all players (in bold: significant correlation *p* < 0.01).

	Pitch		RMS energy		Spectral centroid		Spectral flux	
	*r*	*p*	*r*	*p*	*r*	*p*	*r*	*p*
Arm motions	−0.208	0.393	−0.156	0.523	−0.089	0.718	−0.027	0.913
Knee motions	−0.110	0.655	0.141	0.564	**0.486**	0.035	0.001	0.999

Subsequently, for each player, the correlations between the measured motion angles and the acoustic features were investigated over time (see Table 2 for the detailed correlation coefficients). Positive correlations with the knee motions represent a higher acoustic value when playing with more stretched legs and positive correlations in the arm motions indicate for higher acoustic values when the arms were lifted. As a threshold of interest, only correlation coefficients above Pearson’s *r* > 0.25 and below *r* < −0.25 were considered as meaningful. All these correlations were also significant. Some additional correlations below this threshold were also significant, but were not considered for reasons of clarity.

**Table 2:** Correlation coefficients with significances between the movement trajectories in the motion areas and the acoustic parameters for each player (in bold: *r* > 0.25 or *r* < −0.25).

Players by type	Knee motions								Arm motions							
	Pitch		RMS energy		Spectral centroid		Spectral flux		Pitch		RMS energy		Spectral centroid		Spectral flux	
		*r*	*p*	*r*	*p*	*r*	*p*	*r*	*p*	*r*	*p*	*r*	*p*	*r*	*p*	*r*	*p*
**NoSMP**	*1*	**0.609**	<0.001	**0.373**	<0.001	**0.383**	<0.001	−0.139	<0.001	**−0.289**	<0.001	−0.131	<0.001	**−0.077**	0.025	**0.247**	<0.001
	*2*	**0.607**	<0.001	**0.357**	<0.001	**0.332**	<0.001	0.076	0.02	**0.400**	<0.001	**0.348**	<0.001	**0.177**	<0.001	**−0.094**	0.004
	*3*	**0.445**	<0.001	**0.279**	<0.001	0.097	0,006	0.079	0.025	**0.422**	<0.001	0.035	0.501	0.069	0.021	**−0.404**	<0.001
	*4*	**0.272**	<0.001	0.120	<0.001	0.187	<0.001	−0.248	<0.001	−0.144	<0.001	−0.193	<0.001	−0.110	<0.001	−0.085	0.011
	*5*	−0.026	0.512	0.113	<0.001	−0.071	0.042	**−0.268**	<0.001	**0.443**	<0.001	**0.529**	<0.001	**0.501**	<0.001	−0.061	0.078
**ParmM**	*6*	**0.265**	<0.001	0.110	<0.001	**0.278**	<0.001	−0.145	<0.001	**0.506**	<0.001	**0.304**	<0.001	**0.470**	<0.001	−0.078	0.018
	*7*	0.085	0.018	0.194	<0.001	−0.096	0.006	−0.010	0.775	**0.343**	<0.001	0.246	<0.001	0.165	<0.001	−0.176	<0.001
	*8*	**0.278**	<0.001	0.124	<0.001	−0.078	0.011	−0.205	<0.001	**0.450**	<0.001	0.181	<0.001	**0.464**	<0.001	−0.057	0.062
	*9*	**0.486**	<0.001	**0.590**	<0.001	**0.284**	<0.001	−0.178	<0.001	**0.257**	<0.001	**0.405**	<0.001	0.160	<0.001	−0.087	0.013
**PkneeM**	*10*	0.159	<0.001	0.080	0.016	0.132	<0.001	−0.220	<0.001	**0.319**	<0.001	**0.323**	<0.001	**0.362**	<0.001	0.073	0.027
	*11*	−0.032	0.387	0.127	<0.001	−0.019	0.579	0.034	0.312	**0.361**	<0.001	0.226	<0.001	**0.295**	<0.001	−0.086	0.011
	*12*	**0.326**	<0.001	**0.317**	<0.001	**0.310**	<0.001	0.100	0.002	0.098	0.005	**0.309**	<0.001	0.095	0.004	0.184	<0.001
**LowMP**	*13*	**−0.251**	<0.001	−0.235	<0.001	**−0.294**	<0.001	0.036	0.356	**0.578**	<0.001	**0.596**	<0.001	**0.361**	<0.001	−0.193	<0.001
	*14*	**−0.641**	<0.001	**−0.447**	<0.001	**−0.463**	<0.001	0.033	0.368	**0.595**	<0.001	**0.421**	<0.001	**0.457**	<0.001	−0.179	<0.001
	*15*	**0.693**	<0.001	0.149	<0.001	**0.581**	<0.001	−0.222	<0.001	−0.037	0.322	−0.207	<0.001	0.014	0.681	0.043	0.212
	*16*	−0.206	<0.001	0.026	0.438	−0.222	<0.001	−0.151	<0.001	**0.316**	<0.001	0.046	0.161	0.240	<0.001	**−0.336**	<0.001
	*17*	**−0.349**	<0.001	**−0.299**	<0.001	−0.085	0.015	0.199	<0.001	**0.563**	<0.001	**0.294**	<0.001	−0.008	0.825	−0.154	<0.001
	*18*	0.059	0.089	0.088	0.008	−0.118	<0.001	−0.153	<0.001	**0.779**	<0.001	**0.453**	<0.001	**0.480**	<0.001	**−0.323**	<0.001
	*19*	**0.592**	<0.001	**0.700**	<0.001	**0.281**	<0.001	**−0.332**	<0.001	**0.617**	<0.001	**0.636**	<0.001	**0.581**	<0.001	−0.138	<0.001

Overall, the pitch and the RMS energy values in many cases showed high positive correlations with the knee and the arm motions. As a pattern, both pitch and RMS energy showed a rather common correlation either in the knees or in the arms. For the NoSMP players, however, it was not consistent which motion region correlated most strongly with these acoustic parameters. While for some players the knees correlated highly with the pitch (P1 and P2), other players showed high correlations in the arm motions (P3 and P5) and one player showed rather low correlations in both body regions with a light preference in the knees (P4). Most of the ParmM players showed pronounced correlations with the arm motions (P6, P7 and P8), while it is noteworthy that one player rather used the knee motions in relation to these acoustic parameters even when performing preferably with large arm motions (P9).

Perhaps counterintuitively, the PkneeM motion type mainly showed rather low correlations between knee motions and the investigated acoustic parameters. Only for one of the players, medium correlations could be observed between this motion region and pitch, RMS energy and spectral centroid values. For the other two players with predominant knee motions, however, the connection between arm motions and acoustics seemed to be more pronounced (P10 and P11).

The correlations of the LowMP players in the knee movements showed a very large variation between 0.7 and − 0.64. This seems to be caused by the very low motion ranges in the knees of these players. These correlations are therefore not convincing and have to be considered with caution. The players of this motion type mainly arranged their arm motions with the acoustic parameters pitch and RMS energy. Only one player showed no correlations at all with the arm motions (P15).

Overall, spectral centroid and spectral flux yielded less consistent correlations in all motion types. For only a few players, the correlations reached considerable levels. High correlations with the spectral centroid were found in two players in case of the arm motions (P5 and P19). Additionally, seven players showed medium correlations with the arm motions. For the knee motions, only five players (without the LowMP type) displayed medium correlations.

## Discussion

In this study, ancillary movements of clarinet playing were investigated in their relation to acoustic features during playing. For that, a focus was set to the movements in the arms and the knees. A previous study showed that these are the major motion areas of clarinetists during playing (Weiss et al., 2018). The movement angle trajectories were then analyzed in terms of systematic patterns during the playing and in conjunction with specific audio parameters extracted from the recordings of their performance.

### Visual Movements

The analysis of the visual movements indicated that the players overall performed with rather similar motion behaviors that followed certain musical structures of the piece. The piece is divided into six phrases in which each phrase contains a distinct melodic theme. Between these phrases, the players could breathe in. The knee motions showed that on average the players bent their knees during the phrases and returned to an upright position at the end of each phrase. Even if some players performed slightly different and the players of the LowMP motion type even with nearly no knee movements at all, this particular motion pattern was found as an overall tendency across players and performances. Following the proposition that ancillary movements are often aligned with musical structures (Wanderley et al., 2005; Davidson, 2007), this finding indicates that the specific melodic composition induced the players to perform with this common knee motion behavior.

The recurrence to an upright position at the end of each phrase might serve the purpose to symbolize the end of the melodic part. However, it might also be performed to bring the body into a better position to breathe in. During rapid and effective inhalation, the diaphragm, with the crus attached to the lumbar region of the spine, is pulled down and flattened. The contraction of the diaphragm is accompanied by a contraction of the iliopsoas muscles in the pelvis, which attach to the femur and are associated with flexion of the hip-knee angle. The physiological movement during inhalation is therefore a bending of the hips and knees (Richter, 2014; Spahn, 2015). The inhalation is physiologically preceded by an exhalation at the end of the musical phrase with stretching in the hips and knees. From this perspective, it is reasonable to assume that the observed movements in the knees at the transitions between musical phrases fulfill both an expressive and a physiological function.

The movements of the arms also showed significant patterns across the players. There was a lifting of the arms in the first four phrases at a relatively fixed position, i.e., after two-third of the phrase. In the last two phrases, the arms moved with larger amplitudes than in the previous phrases and showed a rather smooth movement trajectory when the prolonged notes were played. At the end of each phrase, the arms went back to a more neutral position close to the torso. As the movements of the clarinet bell were shown to be related to specific melodic phrasings (Teixeira et al., 2015), these findings indicate that there is also a common motion behavior of the arms in relation to the musical structure.

The velocities of the body movements were calculated to provide an additional value regarding the motions on top of the angle trajectories. A particular expectation was that besides of angular peaks it might also be possible that velocity peaks play a role in individual motion behaviors. However, the velocity values across the piece showed only low variations indicating rather fluent motion performances without large velocity peaks. Due to the low variance, the velocity values showed no significant correlations with the acoustic parameters and were therefore not considered in more detail in the results section. Hence, despite of large amplitudes of motion in the knees and the arms, the velocities were quite similar. This finding might suggest that the players anticipated their movements and prepared them to avoid fast motion actions.

### Acoustic Commonalities

The mean values of the acoustic parameters of all players showed that the first four phrases were played with rather similar dynamics while the last two phrases with the prolonged notes were performed slightly louder. This followed mainly the musical composition of the piece. The last two phrases also show significant differences with regard to the included timbre descriptors, i.e., spectral centroid and spectral flux.

### Multimodal Findings

For the combined analysis of the visual movements and the acoustic parameters, different approaches have been addressed. At first, the differences in the mean values of the acoustic features across the whole performance had been compared between the motions types. The results did not find any significant effects. This indicates that there was no fundamental difference between the performances of the different motion types from acoustic perspective and could lead to the assumption that these ancillary movements are mainly visually intended. However, a limitation of this analysis was the small sample size within each of the motion type groups. Therefore, this finding just provides a rough comparison between the motion types.

The closer look at specific peaks in the movement trajectories however indicated some differences between the motion types. Players of the PkneeM type performed significantly louder when reaching the tipping point of the flexed knees than in extended knee positions. Similarly, players of the ParmM type were louder when playing with arms raised compared to positions with arms alongside the torso. It seems that in these two motions types with predominant movement behavior in the knees or in the arms, the strong movements have an association with the dynamics of the sound. The players may have used these movement peaks to support the acoustic aspects. On the other hand, the acoustic parameter could have been affected by the strong motion action of the knees.

The NoSMP motion type players also showed significant differences in the acoustic features, but only in the timbre characteristics. These acoustic changes were not limited to a single motion area, but were evident in both knee and arm movements. The arm movements were associated with the timbral brightness and higher values were found when the arms were lifted. Therefore, the movement amplitudes seemed to be mainly related to timbre rather than dynamic changes in comparison to the predominant movement types.

In contrast, the detailed correlation analysis showed rather complex results. The correlations between the variances of the body movements and the acoustic parameters across all players indicated that only the motion range of the knees was associated with changes in the spectral centroid. This finding suggests that timbral brightness was higher when the knees were stretched. Bending the knees seems to be associated with a reduction in brightness. This effect can be directly attributed to the typical sound radiation of the clarinet: With a fixed recording microphone position, the change in distance between the clarinet (especially its bell) and the microphone is largest for the PkneeM compared to the other types of movements. Even here, the movements are too small to achieve noteworthy differences in the dynamics, however, they are large enough to cause timbral differences related to the spectral centroid: Due to the fact that the sound radiation of the clarinet becomes more and more directional with increasing frequency, the higher frequencies of the clarinet radiate more directly toward the microphone at some points (with knees outstretched) and more below the microphone at others (with buckled knees; for the sound radiation pattern of the clarinet at higher frequencies, see Meyer, 2009). Due to that effect, the timbral variance cannot be explained as an intentional product of the players movement in those cases.

When looking at the relations between the acoustic parameters and the motion trajectories, the pitch and the RMS energy were most frequently correlated with the visual motions. This suggests that specifically intended ancillary movements were often synchronized with the melodic structure and accompanied by a louder playing. However, the correlation table yielded that for some players both acoustic features were not directly connected. Some showed only correlations with the pitch, but not with the RMS energy. This indicates that the link between visual movements and the melodic trajectory seemed to be stronger than with dynamic changes. Nevertheless, the peak analysis found that especially for the PkneeM motion type, when performing a strong knee bending, the loudness was significantly higher compared to a more upright position. This however, could not be observed in the detailed correlation analysis. It therefore seems that this particular movement behavior was only relevant at strong peak levels and not during the whole playing. Most interestingly, two of the three PkneeM players synchronized their arm movements more with the acoustic features similar to the players of the other motion types. This might suggest that the knee behavior in this motion type seemed to follow an expression more visually intended.

The finding that higher notes were preferably played with more straightened legs overall, leads to the assumption that the knee movements form some sort of visual assistance of the notes. As the melodic progression in each phrase consists of low notes at the beginning followed by higher notes toward the end, the movements of the knees seemed to follow these melodic structures. This might support the idea of a musical gesture (Godøy and Leman, 2010) demonstrating a music-induced connection between movement and melody. However, since this study only used one piece of music, which has a particular musical structure, a specific relationship can only be presumed.

The players of the LowMP motion type performed with only very small knee movements. This was particularly difficult for the movement peak analysis, as the turning points were rather sparse and very close to the threshold of the detection process. With that, the validity of the correlations with the knee movements was supposedly rather low. It is therefore assumed that these players did not use the knee movements for any specific purpose but for standing in an upright position providing optimal playing and breathing conditions. In contrast, the arms were very much correlated with the acoustic features. Even though they did not perform as pronounced arm movements as other motion types, they too connected this motion behavior considerably close with the acoustics.

In summary, the analysis suggests that the visual movements of the arms and knees contained clear expressive components that were related to the musical structure. During the performance, they followed melodic elements but also were consistent across players at particular musical positions, i.e., transitions and at the end, confirming previous findings (Thompson and Luck, 2012). Moreover, those movements were not only of ancillary character but contained certain aspects of instrumental movements. The players seemed to perform a particular adaptation in the use of the body movements between addressing a musical gesture and providing a necessary physiological posture for playing and breathing. These different elements of movements interact with each other but at certain musical requirements, the physiological movements become more prominent.

## Limitations

As has been mentioned before, the small sample sizes within the motion types are potentially problematic. However, the study in Weiss et al. (2018) was able to identify clear differences between the four player types regarding the movement areas of the knees and the arms. In this current study, it was shown that there was no clear relation between those distinct motion types and acoustic features. The results rather indicate that the relations between the visual and acoustic performance seemed to be were very individual for each player.

A limitation with regard to the detailed correlation analysis between the measured motion angles and the acoustic features per player (e.g., Table 2), is that the individual measurements over time are not independent since both types of measurements (motion and acoustic) are time series data that can be autocorrelated to some degree (Dean and Dunsmuir, 2016). Moreover, it has to be kept in mind that many combinations (of player, audio feature and motion angle) are included in this detailed analysis, so the significance of each individual correlation has to be interpreted with caution. A further analysis could include a linear mixed modeling approach in order to better account for those effects.

Since the study investigated a wind instrument, the strong aspect of breathing should be taken into account. The main regions of ancillary movements were found in the upper body parts (i.e., torso, arms, and shoulders) and the lower body (i.e., the legs). Both provide a main contribution to the respiration process as the respiratory physiological movements in wind instruments form a specific part of instrumental movements. It would therefore be interesting to learn more about the physiological relationship between breathing and arm movements, for example, in order to be able to describe the overlaps between expressive and physiological movements in a more differentiated way.

In addition to the body areas knees and arms, a particular movement of players is the side-to-side swaying. Since the previous study on the visual movements (Weiss et al., 2018) found that this was performed very individually and without certainty, this movement area was not taken into account here. However, it would be interesting to compare different playing situations of clarinetists regarding visual and audio aspects. In particular, a comparison between the standing and the sitting position could be interesting because in orchestras, the players mostly perform in a sitting position. This would follow the findings in trombone players where abdominal muscle activity was significantly reduced when sitting (Price et al., 2014).

## Conclusion

The results of this study showed that clarinetists used their body movements very individually, but played with rather similar motion patterns during certain musical phrases, such as at transitions between melodic parts. The relationship between these movements with changes in the sound was also found to be rather individual. Only a few general associations could be identified. It was found that on average players with large movements in the arms or the knees connected these motions mainly with the dynamics of the sound.

Regarding the body movements, the players seem to perform in two different ways: with ancillary movements and with instrumental movements simultaneously following the goal of expressing musical intentions and providing a proper posture for playing and breathing. These two aspects of movements interact with each other according to the musical structure.

## Data Availability Statement

The raw data supporting the conclusions of this article will be made available by the authors, without undue reservation.

## Ethics Statement

The studies involving human participants were reviewed and approved by Ethics Committee of the University Clinic Freiburg. The patients/participants provided their written informed consent to participate in this study.

## Author Contributions

MN did mainly the data collection. IC-E, CR and MN performed the statistical analyses. All authors contributed to the article and approved the submitted version.

## Funding

We acknowledge support by the Open Access Publication Fund of the University of Freiburg. The article processing charge was partially funded by the Open Access Office of the Vienna University Library.

## Conflict of Interest

The authors declare that the research was conducted in the absence of any commercial or financial relationships that could be construed as a potential conflict of interest.

## Publisher’s Note

All claims expressed in this article are solely those of the authors and do not necessarily represent those of their affiliated organizations, or those of the publisher, the editors and the reviewers. Any product that may be evaluated in this article, or claim that may be made by its manufacturer, is not guaranteed or endorsed by the publisher.

## References

[ref1] Ackermann D. Böhm C. Brinkmann F. Weinzierl S. (2019). The acoustical effect of musicians’ movements During musical performances. Acta Acust. unit. Acust. 105, 356–367. doi: 10.3813/AAA.919319

[ref2] Cadoz C. Wanderley M. M. (2000). “Gesture - Music” in Trends in Gestural Control of Music. eds. Wanderley M. M. Battier M. (Ircam: Ircam-Centre Pompidou).

[ref3] Chang A. Kragness H. E. Livingstone S. R. Bosnyak D. J. Trainor L. J. (2019). Body sway reflects joint emotional expression in music ensemble performance. Sci. Rep. 9:205. doi: 10.1038/s41598-018-36358-4, PMID: 30659220 PMC6338747

[ref4] Cohen J. (1988). Statistical Power Analysis for the Behavioral Sciences. 2nd Edn.. Hillsdale, N.J: L. Erlbaum Associates.

[ref5] Coorevits E. Maes P.-J. Six J. Leman M. (2020). The influence of performing gesture type on interpersonal musical timing, and the role of visual contact and tempo. Acta Psychol. 210:103166. doi: 10.1016/j.actpsy.2020.103166, PMID: 32919094

[ref6] Dahl S. Bevilacqua F. Bresin R. (2010). “Gestures in Performance” in Musical Gestures: Sound, Movement, and Meaning. eds. Godøy R. I. Leman M. (New York: Routledge), 48–80.

[ref7] Dahl S. Friberg A. (2007). Visual perception of expressiveness in musicians’ body movements. Music. Percept. 24, 433–454. doi: 10.1525/mp.2007.24.5.433

[ref8] Davidson J. W. (2007). Qualitative insights into the use of expressive body movement in solo piano performance: a case study approach. Psychol. Music 35, 381–401. doi: 10.1177/0305735607072652

[ref9] Davidson J. W. (2011). “Movement and collaboration in musical performance” in Oxford Handbook of Music Psychology. eds. Hallam S. Cross I. Thaut M. (London: Oxford University Press), 364–376.

[ref10] Davidson J. W. (2012). Bodily movement and facial actions in expressive musical performance by solo and duo instrumentalists: two distinctive case studies. Psychol. Music 40, 595–633. doi: 10.1177/0305735612449896

[ref11] Davidson J. W. Broughton M. C. (2016). “Bodily mediated coordination, collaboration, and communication in music performance,” in The Oxford Handbook of Music Psychology. eds. Hallam S. Cross I. Thaut M. H.. 2nd Edn. (New York, NY, US: Oxford University Press), 573–595.

[ref12] Dean R. T. Dunsmuir W. T. M. (2016). Dangers and uses of cross-correlation in analyzing time series in perception, performance, movement, and neuroscience: The importance of constructing transfer function autoregressive models. Behav. Res. Ther. 48, 783–802. doi: 10.3758/s13428-015-0611-2, PMID: 26100765

[ref13] Demos A. P. Chaffin R. Logan T. (2018). Musicians body sway embodies musical structure and expression: a recurrence-based approach. Music. Sci. 22, 244–263. doi: 10.1177/1029864916685928

[ref14] Gabrielsson A. Juslin P. N. (2003). “Emotional expression in music” in Handbook of Affective Sciences Series in Affective Science (New York, NY, US: Oxford University Press), 503–534.

[ref15] Glowinski D. Mancini M. Cowie R. Camurri A. Chiorri C. Doherty C. (2013). The movements made by performers in a skilled quartet: a distinctive pattern, and the function that it serves. Front. Psychol. 4:841. doi: 10.3389/fpsyg.2013.00841, PMID: 24312065 PMC3826428

[ref16] Godøy R. I. Leman M. (eds.) (2010). Musical Gestures: Sound, Movement, and Meaning. New York: Routledge.

[ref18] Gritten A. King E. (2016). New Perspectives on music and Gesture. London: Routledge Available at: https://www.taylorfrancis.com/books/e/9781315598048 (Accessed December 29, 2021).

[ref19] Jensenius A. R. (2018). “Methods for Studying Music-Related Body Motion” in Springer Handbook of Systematic Musicology Springer Handbooks. ed. Bader R. (Berlin, Heidelberg: Springer Berlin Heidelberg), 805–818.

[ref20] Jensenius A. R. Wanderley M. M. Godøy R. I. Leman M. (2010). “Musical gestures: concepts and methods in research” in Musical Gestures: Sound, Movement, and Meaning. eds. Godøy R. I. Leman M. (New York, NY: Routledge), 12–35.

[ref21] Juslin P. N. Timmers R. (2010). “Expression and communication of emotion in music performance” in Handbook of Music and Emotion: Theory, Research, Applications Series in Affective Science (New York, NY, US: Oxford University Press), 453–489.

[ref22] Lartillot O. Toiviainen P. Eerola T. (2008). “A Matlab toolbox for music information retrieval” in Data Analysis, Machine Learning and Applications Studies in Classification, Data Analysis, and Knowledge Organization. eds. Preisach C. Burkhardt H. Schmidt-Thieme L. Decker R. (Berlin, Heidelberg: Springer), 261–268.

[ref23] Meyer J. (2009). Acoustics and the Performance of Music. New York, NY: Springer New York.

[ref24] Nusseck M. Wanderley M. M. (2009). Music and motion—how music-related ancillary body movements contribute to the experience of music. Music. Percept. 26, 335–353. doi: 10.1525/mp.2009.26.4.335

[ref25] Nusseck M. Wanderley M. M. Spahn C. (2018). “Body Movements in Music Performances: The Example of Clarinet Players” in Handbook of Human Motion. eds. Müller B. Wolf S. I. (Cham: Springer International Publishing), 1789–1802.

[ref26] Platz F. Kopiez R. (2012). When the eye listens: A Meta-analysis of how audio-visual presentation enhances the appreciation of music performance. Music. Percept. 30, 71–83. doi: 10.1525/mp.2012.30.1.71

[ref27] Price K. Schartz P. Watson A. H. (2014). The effect of standing and sitting postures on breathing in brass players. Springerplus 3:210. doi: 10.1186/2193-1801-3-210, PMID: 25279272 PMC4167884

[ref28] Richter B. (2014). Die Stimme: Grundlagen, künstlerische Praxis, Gesunderhaltung. 2nd Edn. Leipzig: Henschel.

[ref29] Spahn C. (2015). Musikergesundheit in Der Praxis: Grundlagen, Prävention, Übungen. Leipzig: Henschel.

[ref30] Teixeira E. C. F. Loureiro M. A. Yehia H. C. (2018). Expressiveness in music From a multimodal perspective. Music. Percept. 36, 201–216. doi: 10.1525/mp.2018.36.2.201

[ref31] Teixeira E. C. F. Yehia H. C. Loureiro M. A. (2015). Relating movement recurrence and expressive timing patterns in music performances. J. Acoust. Soc. Am. 138, EL212–EL216. doi: 10.1121/1.4929621, PMID: 26428815

[ref32] Thompson M. R. Luck G. (2012). Exploring relationships between pianists’ body movements, their expressive intentions, and structural elements of the music. Music. Sci. 16, 19–40. doi: 10.1177/1029864911423457

[ref33] Tsay C.-J. (2013). Sight over sound in the judgment of music performance. Proc. Natl. Acad. Sci. U. S. A. 110, 14580–14585. doi: 10.1073/pnas.1221454110, PMID: 23959902 PMC3767512

[ref34] Vines B. Krumhansl C. Wanderley M. Levitin D. (2006). Cross-modal interactions in the perception of musical performance. Cognition 101, 80–113. doi: 10.1016/j.cognition.2005.09.003, PMID: 16289067

[ref35] Wanderley M. M. Battier M. (2000). Trends in Gestural Control of Music. Paris: IRCAM, Centre Pompidou.

[ref36] Wanderley M. M. Vines B. W. Middleton N. McKay C. Hatch W. (2005). The musical significance of clarinetists’ ancillary gestures: an exploration of the field. J. New Music Res. 34, 97–113. doi: 10.1080/09298210500124208

[ref37] Weiss A. E. Nusseck M. Spahn C. (2018). Motion types of ancillary gestures in clarinet playing and their influence on the perception of musical performance. J. New Music Res. 47, 129–142. doi: 10.1080/09298215.2017.1413119

[ref38] Wöllner C. (ed.) (2017). Body, Sound and Space in Music and Beyond: Multimodal Explorations. New York: Routledge.

