# Supplementary material for: Associations Between Ancillary Body Movements and Acoustic Parameters of Pitch, Dynamics and Timbre in Clarinet Playing
Source: Front Psychol. 2022 Jul 13;13:885970. doi: 10.3389/fpsyg.2022.885970 (PMC9330450; doi:10.3389/fpsyg.2022.885970)
Supplement: Supplementary file 1 [file Data_Sheet_1.PDF]

Supplementary Material

1 Supplementary Data

The following supplementary material contains the note of the piece the clarinetists were performing.

1.1 Notes of the piece

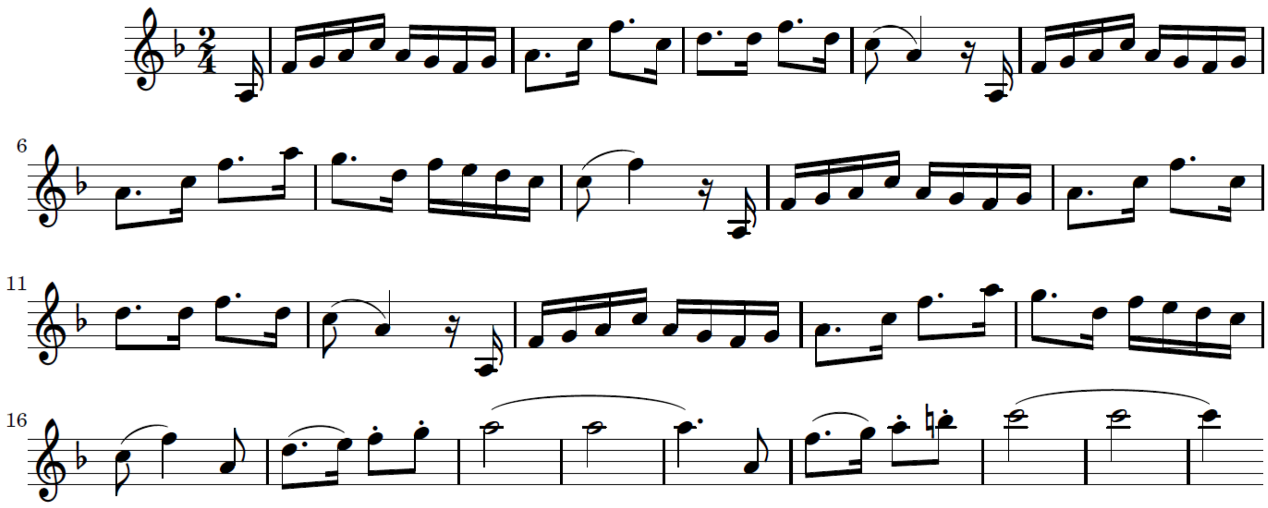

Supplementary Figure 1. Notes of Mendelssohn's 3rd Symphony ("Scottish Symphony", Op. 56, the first 24 bars of the 2nd movement "Vivace" in A minor). Created with LilyPond Score Editor.
